# Subject-Specific 3D Models to Investigate the Influence of Rehabilitation Exercises and the Twisted Structure on Achilles Tendon Strains

**DOI:** 10.3389/fbioe.2022.914137

**Published:** 2022-07-06

**Authors:** Alessia Funaro, Vickie Shim, Marion Crouzier, Ine Mylle, Benedicte Vanwanseele

**Affiliations:** ^1^ Human Movement Biomechanics Research Group, KU Leuven, Leuven, Belgium; ^2^ Auckland Bioengineering Institute, University of Auckland, Auckland, New Zealand

**Keywords:** achilles tendon, fascicle twist, sub-tendon morphology, tendon strain, finite element modeling, rehabilitation exercises

## Abstract

The Achilles tendon (AT) is the largest tendon of the human body and has a primary role in locomotor activities. The complex structure of the AT includes twisting of three sub-tendons, non-uniform tissue deformations and differential triceps surae muscle forces. The main aim of this study was to investigate the impact of commonly used rehabilitation exercises (walking on heels, walking on toes, unilateral heel rise, heel drop with extended knee and heel drop with the knee bent) and different twists on AT strains. 3D freehand ultrasound based subject-specific geometry and subject-specific muscle forces during different types of rehabilitation exercises were used to determine tendon strains magnitudes and differences in strains between the sub-tendons. In addition, three Finite Element models were developed to investigate the impact of AT twist. While walking on heels developed the lowest average strain, heel drop with knee bent exhibited the highest average strain. The eccentric heel drop resulted in higher peak and average strain, compared to concentric heel rise for all the three models. The isolated exercises (heel rise and heel drop) presented higher average strains compared to the functional exercises (walking tasks). The amount of twist influences the peak strains but not the average. Type I consistently showed highest peak strains among the five rehabilitation exercises. The ranking of the exercises based on the AT strains was independent of AT twist. These findings might help clinicians to prescribe rehabilitation exercises for Achilles tendinopathy based on their impact on the AT strains.

## 1 Introduction

The Achilles tendon (AT) plays a major role during weight-bearing locomotor activities by sustaining loads up to 7.7 times bodyweight (Komi et al., 1992) and transfers the generated muscle force from the triceps surae to the calcaneus. Despite being the strongest and thickest tendon of the human body (Maffulli, 1999), the AT is very susceptible to injuries. Achilles tendinopathy is one of the most common foot and ankle overuse injuries (Sobhani et al., 2013), with a prevalence of up to 9.5% and 11.8% in athletic (Lopes et al., 2012) and non-athletic populations (Albers et al., 2016), respectively.

The AT is mechanosensitive, so the exposure to loading leads to changes in its mechanical properties. Loading of the tendon causes complex internal tissue strains which, in turn, induces tendon remodeling. Wang et al. (2015) demonstrated that a certain loading regime might create a proper (internal) mechanical environment, able to reverse early-stage pathological changes in Achilles tendinopathy patients. Consequently, insights into the effect of these exercises on the tendon strain can serve as a guide for progression in rehabilitation, since an appropriate biomechanical environment can facilitate the rehabilitation process of the AT through an optimal loading dose.

Currently, exercise-based therapy with load management is the treatment of choice in clinical practice, to reduce aggravating loads and to introduce pain-relieving loads (Cook and Purdam, 2014). Different rehabilitation protocols were proposed including a variety of eccentric exercises (Alfredson et al., 1998), a combination of eccentric and concentric exercises (Silbernagel et al., 2001) and a combination of more functional exercises (Mascaró et al., 2018). However, there is still little clinical or mechanistic evidence for isolating the eccentric component (Malliaras et al., 2013). In addition, up to 40% of the patients don’t respond to the classical treatment schemes (Maffulli et al., 2008) and it remains unknown if these rehabilitation programs provide an optimal stimulus for healing (Lysholm and Wiklander, 1987) or they are just pain relievers. The progression of exercises is currently mostly based on the subjective feeling of pain rather than the objective tendon loading, which is the driving factor for tissue regeneration. Previous studies attempted to rank the rehabilitation exercises according to AT load. Recently, studies by Baxter et al. (2021) and Devaprakash et al. (2022) developed an exercise progression that incrementally increases AT load. However, in both studies, the AT loading was estimated by external measures, with motion capture in conjunction with ground reaction force data or surface electromyography, which don’t account for the tendon geometry or material properties and cannot describe the internal tendon load. During force production, the tendon strain is governed by the muscle forces but also by the 3D geometry of the AT, which is complex (Edama et al., 2016; Pękala et al., 2017). Tendon fibers originate from triceps surae: the soleus (SOL) and the two heads (medialis, GM and lateralis, GL) of the gastrocnemius muscle, forming three individual sub-tendons (Pękala et al., 2017). Based on the degree of twist, three types of twisted structures have been identified: Type I (least), Type II (moderate) and Type III (extreme) (Pękala et al., 2017). Since the AT arises from three muscles, it will be subjected to three muscles forces and therefore behaves as three partially independent sub-tendons, leading to significant sliding between adjacent sub-tendons. This sliding has been previously reported *in vivo* within healthy AT using ultrasound imaging (Bogaerts et al., 2017), and is thought to reduce the stress within the tendon (Thorpe et al., 2015).

Finite element (FE) models of the AT allow us to investigate the effects of variations in tendon geometry and material properties on local tendon stress and strain. Handsfield et al. (2017) demonstrated that the amount of intra-tendon sliding and tendon twist plays a role on the relationship between muscle forces and tendon behaviour. Shim et al. (2018) further showed that the AT experiences non-uniform tissue deformation between tendon regions when sub-tendons geometry and tendon twist were included in their FE model. This was further confirmed by the fact that varying overall tendon twist in such models showed to impact tendon fascicle length, strain and energy storage (Knaus and Blemker, 2021). The main limitation of these previous studies is that the three sub-tendons were not included in the AT models (Hansen et al., 2017) or, if included, only two sub-tendons were considered (soleus and gastrocnemius) (Shim et al., 2019; Handsfield et al., 2020). In studies that considered the three sub-tendons (Handsfield et al., 2017; Knaus and Blemker, 2021; Yin et al., 2021), the focus was on the analysis of more static exercises. To compare the effect of typical exercises from rehabilitation protocols for Achilles tendinopathy, one needs to use a FE model which includes all three sub-tendons, subject-specific geometry and muscle forces.

Therefore, the aim of this study was to develop and implement FE models including subject-specific geometry and muscle forces during a selection of common rehabilitation exercises to investigate the different types of exercises and the effect of twist on the AT strain. Accordingly, we included exercises to compare eccentric vs. concentric, isolated vs. functional and exercises variation with differential muscle forces. Then, investigating the influence of the type of twist on AT strains could provide insights into potential reasons for subject-specific responses to treatment. Because the different executions of the exercises are expected to redistribute the muscle forces, we hypothesized that they would change the tendon strains. Specifically, we hypothesized that the ranking of the exercises would start from eccentric and concentric exercises, moving towards more functional exercises, as recommended by previous rehabilitation protocols (Mascaró et al., 2018). We also hypothesized that an increase in the degrees of tendon twist would reduce the tendon strain.

## 2 Materials and Methods

A subject-specific geometry of the free tendon as well as the triceps surae muscle forces during the rehabilitation exercises were obtained from one female participant (age = 29 years, weight = 56 kg, height = 174 cm). The participant did not report any (previous) injuries to the AT or foot and ankle complex, nor a systemic disease affecting the collagenous tissue and provided written informed consent before participation.

### 2.1 Generic Model Geometry

To allow the consistent definition of the three sub-tendons within subject-specific FE models, we created first a generic template mesh. This mesh was generated from an initial geometry, obtained by segmentation of images from one healthy male subject (age = 22 years, weight = 64 kg, height = 180 cm) recorded by 3DfUS images, defining the outer geometry of the tendon. Three different types of twist were then created using Materialise 3-matic (Materialise NV, Leuven, Belgium). The tendon model was divided into three sub-tendons. Different twist angles were applied that resulted in three twisted structures that corresponded with the classification of the AT twist (Type I, Type II, and Type III) described by Pękala et al. (2017). The sub-tendons geometries were meshed into 8-nodes hexahedral solid elements. A mesh convergence study was performed to refine the mesh until the Principal Effective Lagrange strains reached an asymptote.

### 2.2 Constitutive Models

The tendon models were represented as an incompressible, transversely isotropic hyperelastic material (Weiss et al., 1996). The uncoupled strain energy function can be written as in Eq. 1:

$$\Psi =F_{1}\left({\tilde{I}}_{1},{\tilde{I}}_{2}\right)+F_{2}\left(\tilde{\lambda }\right)+\frac{K}{2}{\left[\ln\left(J\right)\right]}^{2}$$ (1)

Here, ${\tilde{I}}_{1}$ and ${\tilde{I}}_{2}$ are the first and second invariants of the deviatoric version of the right Cauchy-Green deformation tensor. $\tilde{\lambda }$ is the deviatoric part of the stretch along the fiber direction, and J=det(F) is the Jacobian of the deformation. This strain energy density function consists of two parts: *F*_*1*_ represents the material response of the isotropic ground substance matrix, while *F*_*2*_ represents the contribution from the collagen fibers. *F*_*1*_ was described as a Neo-Hookean model and it was equal to $\frac{C_{1}\left(I_{1}-3\right)}{2}$ . The resulting fiber stress from the fibers was expressed using the piecewise function in Eq. 2:

$$\tilde{\lambda }\frac{\partial F_{2}}{\partial \tilde{\lambda }}=\{\begin{array}{cc} 0 & \tilde{\lambda }\leq 1\\ C_{3}\left(e^{C_{4}\left(\tilde{\lambda }-1\right)}-1\right) & 1<\tilde{\lambda }<\\ C_{5}\tilde{\lambda }+C_{6} & \tilde{\lambda }\geq \lambda_{m} \end{array}\lambda_{m}$$ (2)

Here, $\lambda_{m}\;$ is the strain at which the fibers are straightened, *C*_*3*_ is the scaling of the exponential stress, *C*_*4*_ is the rate of the uncrimping of the fibers and *C*_*5*_ is the Young’s modulus of the straightened fibers. *C*_*6*_ is determined from the requirement that the stress is continuous at $\lambda_{m}$ . The values of the material properties that were used for the models were obtained from previous studies (Hansen et al., 2017; Knaus and Blemker, 2021) and can be found in the Supplementary Material. The constitutive models implemented in FEBio Studio (Maas et al., 2012) as “*trans iso Mooney-Rivlin*” were used in this study.

### 2.3 Model Fascicles

The model sub-tendons fascicles were defined in FEBio (Maas et al., 2012): a local fiber direction (*a*_*0*_) was defined for each element to represent the tendon fascicle structure (Knaus and Blemker, 2021). For each 3D sub-tendon, fibers were directed from the proximal cross-section to the distal cross-section. In this way, the local fibers resulted in different fascicle twist angles in each model, as described in the literature (Pękala et al., 2017).

### 2.4 Subject-Specific Volume Reconstruction and Segmentation From Freehand Three-Dimensional Ultrasound

A conventional 2D ultrasound machine with a linear transducer (ArtUS, UAB Telemed, Vilnius, Lithuania) was used to record images of the AT. The ultrasound machine was combined with an optical motion tracking system (Optitrack NaturalPoint, United States) to generate a 3D reconstruction of the AT during rest. During the acquisition, the participant was positioned prone with a fixated foot and a neutral ankle angle. The 2D ultrasound images were transformed into the global coordinate system using 3D Slicer (Version 4.11.20210226) to create a reconstructed 3D volume. After computing a 3D volume reconstruction, the AT was manually outlined using the reconstructed 2D images, and the corresponding borders were interpolated to generate a 3D mesh of the tendon. The tendon geometry of the subject-specific mesh was reconstructed.

### 2.5 Subject-Specific Muscle Force Estimation

The participant came to the Movement and posture Analysis Laboratory Leuven (Belgium) for a single session and started with a 5-min warm-up on a stationary bicycle. The participant completed five repetitions of five rehabilitation exercises in a randomized order: walking on heels (heel walk), walking on toes (toe walk), unilateral heel rise (unirise), heel drop with extended knee (unidrop) and heel drop with knee bent (unidrop bent). Description of the execution of the exercises can be found in the Supplementary Material. In between trials, the participant was given a minimum of 30 s of rest before moving on to the next trial.

An extended Plug-in Gait marker set including 34 retroreflective markers, of which the trajectories were recorded using ten infrared cameras (Vicon, Oxford Metrics, Oxford, United Kingdom) at a sampling rate of 150 Hz, were placed on anatomical landmarks to obtain kinematic data. Ground reaction force data was measured from the participant’s dominant leg using a force plate embedded in the walkway. A modified generic musculoskeletal model (OpenSim gait2392 model) (Delp et al., 1990) with 6 degrees of freedom and 43 Hill-type muscle-tendon actuators per leg was scaled in OpenSim 3.3 (OpenSim, Stanford, CA, United States) and joint kinematics were then computed using a Kalman Smoothing algorithm (De Groote et al., 2008). Next, an inverse dynamic approach was used to calculate the joint moments. SOL, GM, and GL muscle forces were then estimated using a dynamic optimization method by minimizing the sum of squared muscle activations (De Groote et al., 2016). The muscle forces at the time of peak total muscle force during each exercise were used as boundary conditions in the FE model (Table 1).

**TABLE 1:** The estimated peak muscle forces during the rehabilitation exercises, applied on each sub-tendon and used as boundary conditions for the finite element analysis. The unit of the muscle forces is Newton (N).

Exercise	GM force (N)	GL force (N)	SOL force (N)
Heel walk	108.8	78.9	180.2
Toe walk	328.4	154.7	702.3
Unirise	721.4	164.1	576.0
Unidrop	982.8	422.5	91.4
Unidrop Bent	351.7	84.6	1589.6

### 2.6 Creation of a Subject-Specific FE Model With Free-form Deformation

**FIGURE 1:**
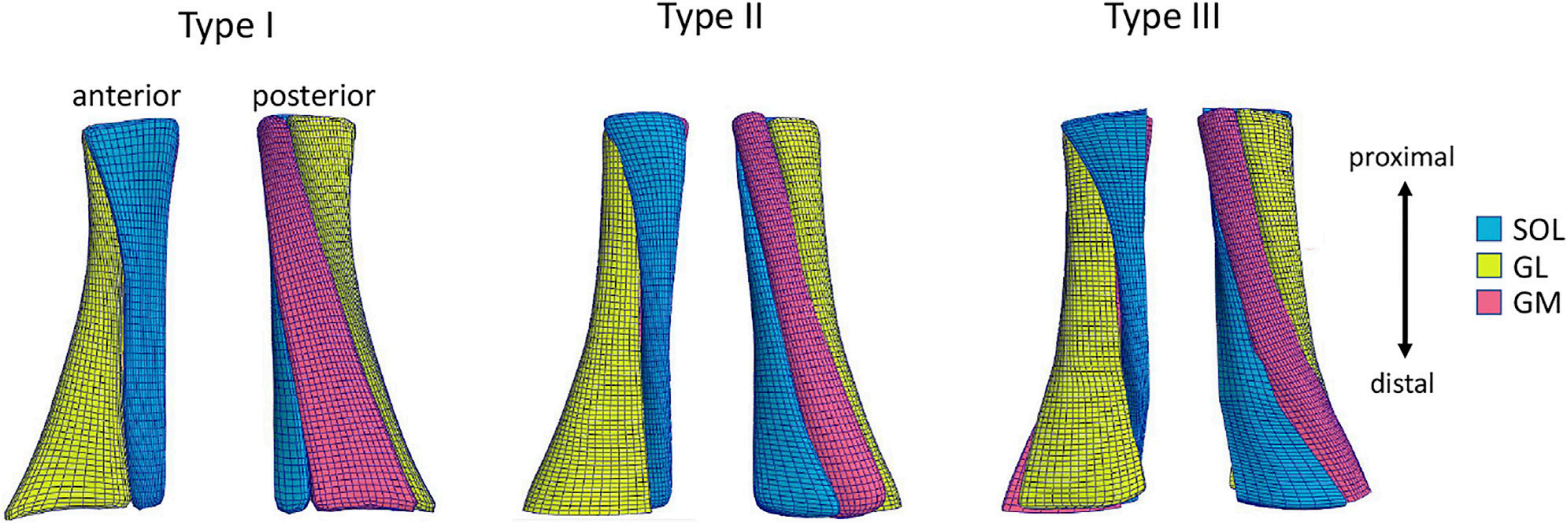
Anterior and posterior view of the Achilles free tendon geometries representing the three types of twist: Type I (least), Type II (moderate), Type III (extreme). Each 3D tendon model was divided in three sub-tendons, each one arising from one of the three triceps surae muscles: the soleus (SOL) and the two heads (medialis, GM and lateralis, GL) of the gastrocnemius muscle.

Subsequently, the template mesh containing three sub-tendons was customized to the subject-specific geometry obtained with the subject’s 3DfUS image. We used the free-form deformation method (Fernandez et al., 2018), which morphs an underlying mesh by embedding it inside a host mesh. This allows the nodes on the external surface of the given mesh to match the subject’s geometry while the internal nodes of the mesh are also deformed using the same transformation. In this way, it was possible to obtain three subject-specific free AT geometries, representing the three twists described in the literature (Pękala et al., 2017) (Figure 1). The length of the 3D geometry was 40 mm and the volume was 1,136 mm^3^ on average between twisted geometries.

### 2.7 Subject-Specific Model Boundary Conditions

The contact between the three sub-tendons was defined as frictionless sliding (Knaus and Blemker, 2021). The distal end of the three models was fixed. Different rehabilitation exercises were simulated by applying muscle forces (Table 1) as nodal loads to the proximal faces of each sub-tendon. The nodal displacements were constrained to move only in the distal-proximal direction to mimic the constraints provided by fascia tissues that hold the sub-tendons together.

### 2.8 Strain Analysis

**FIGURE 2:**
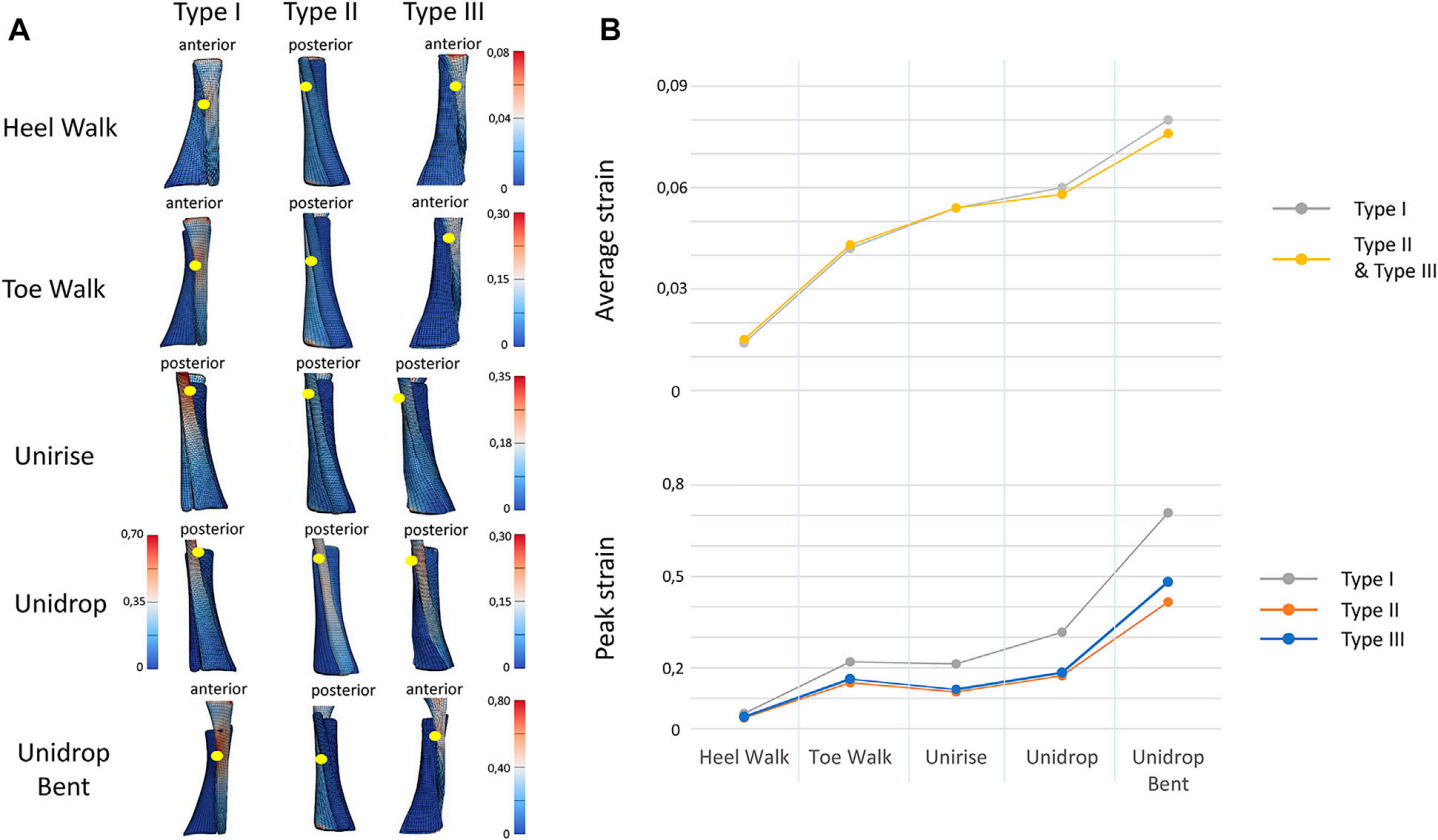
**(A)**. View of the distribution of the maximum Lagrange strain for the three types of twist and for all the five rehabilitation exercises. The yellow dots indicate the location of the peak strain. **(B)** Trend of the peak and average of the maximum Lagrange strain for all the types of twist and all the rehabilitation exercises, in the mid-portion of the tendon models. In average strain, Type I and Type II strain are described with the same line, since the values are the same.

**FIGURE 3:**
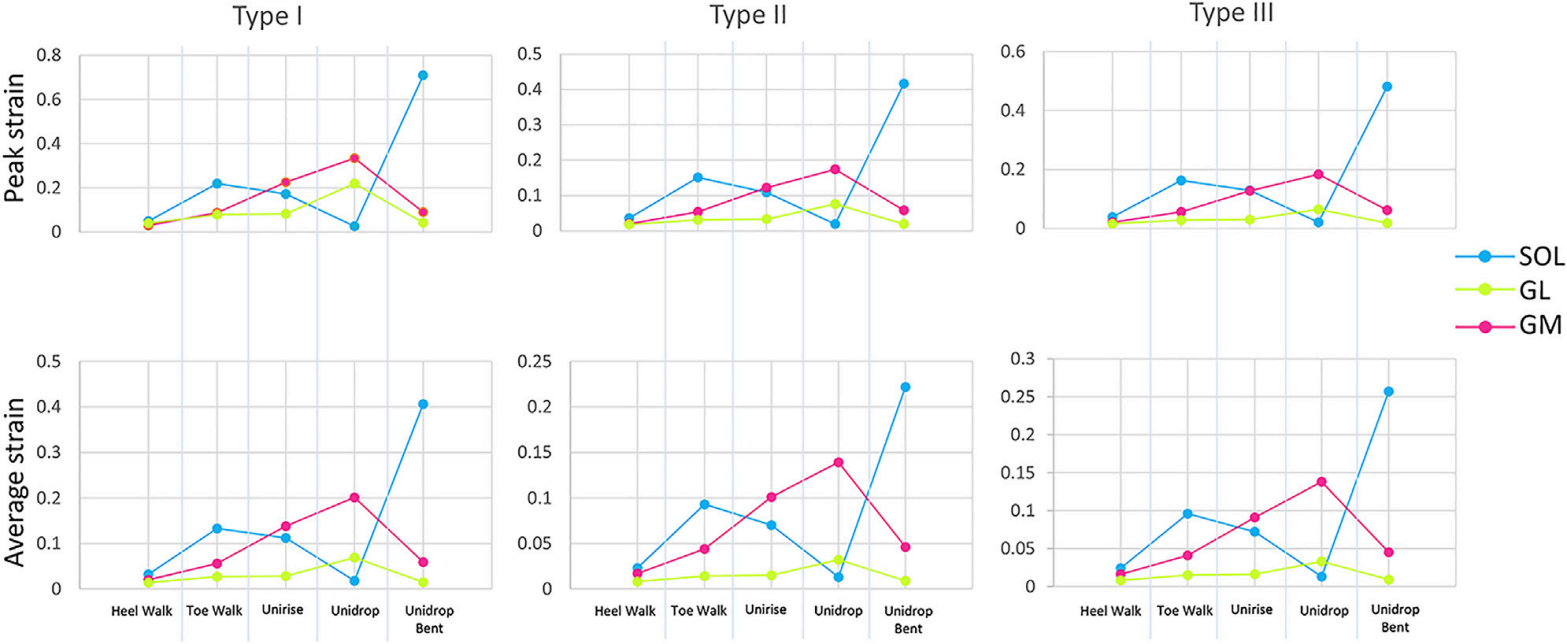
Peak and average of the maximum Lagrange strain at the mid-portion of each sub-tendon, soleus (SOL), gastrocnemius medialis (GM) and gastrocnemius lateralis (GL), for all the five rehabilitation exercises and twist.

To quantitatively analyze strain distribution patterns during the different exercises, two different analysis were performed. Firstly, we examined the peak and average of the maximum principal strain, in the mid-portion of the tendon (defined as the center third of the AT models), to identify the differences between different types of rehabilitation exercises (Figure 2B). We also analyzed the peak and average of the maximum principal strain in the mid-portion of each sub-tendon, to examine the non-uniform deformations of such sub-tendons (Figure 3). Secondly, we examined the distribution of the maximum principal strain and location of the peak maximum principal strain in the whole free tendon, to characterize the overall strain patterns between different twist types and exercises (Figure 2A).

## 3 Results

### 3.1 Effect of Task Type on Tendon Strains

#### 3.1.1 The Effect of Bending the Knee During Unilateral Heel Drop

Bending the knee during the heel drop exercise (unidrop bent) redistributed the muscle force and increased the peak strain by 0.392 for Type I, 0.242 for Type II and 0.298 for Type III, and the average strain by 0.046 for Type I, 0.018 for Type II and 0.02 for Type III, in the mid-portion (Figure 2B). Bending the knee also had an effect on the difference in tendon strain between sub-tendons in the mid-portion. SOL sub-tendon showed the greatest strains compared to GM and GL sub-tendons when the knee was bent. During unidrop, GM sub-tendon showed the largest strains followed by SOL and GL sub-tendons (Figure 3). This was observable for all types of twists.

#### 3.1.2 Eccentric Versus Concentric Exercises

Unidrop exercise resulted in higher peak (Type I: 0.317 vs. 0.213, Type II: 0.174 vs. 0.121, Type III: 0.184 vs. 0.129) and average strain (Type I: 0.096 vs. 0.088, Type II: 0.058 vs. 0.054, Type III: 0.06 vs. 0.054), in the mid-portion, compared to concentric unirise for all the three models (Figure 2B). The type of exercises also had an effect on difference in tendon strains between sub-tendons. During both unirise and unidrop, the largest peak and average strains were observed in the GM sub-tendon for all types of twist, but the difference of the strains between GM and SOL sub-tendons increased for the eccentric heel drop (Figure 3).

#### 3.1.3 Isolated Versus Functional Exercises

The isolated exercises (unirise and unidrop) presented higher average strains compared to the functional exercises (walking tasks), in the mid-portion of the AT (Figure 2B). However, toe walk showed higher peak strain than unirise, for all types of twist (Type I: 0.220 and 0.213, Type II: 0.151 and 0.121, Type III: 0.163 and 0.129, peak strains, for toe walk and unirise, respectively). The type of exercises had also an effect in the difference in tendon strains between sub-tendons, in the mid-portion. While during the functional exercises the highest strains were observed in the SOL sub-tendon, during the isolated exercises the highest strains were in the GM sub-tendon. The difference in strains of the sub-tendons increased going from the functional exercises to the isolated ones (Figure 3).

### 3.2 Ranking of the Rehabilitation Exercises Based on Tendon Strains

The ranking of the exercise is independent of the twist of the tendon. Starting from the lowest average strain in the mid-portion of the AT models, the ranking of the rehabilitation exercises was: heel walk, toe walk, unirise, unidrop and unidrop bent, which exhibited the highest average strains (Figure 2B).

### 3.3 Effect of the Twist on Tendon Strains and Peak Value Location

The amount of twist influences the peak strains but not the average strains nor the ranking of the rehabilitation exercises. Type I consistently showed higher peak strains than the other two types, regardless of the type of rehabilitation exercise. Type II showed the lowest peak strains, for all the rehabilitation exercises. The location of the peak strain differed among the three different types of twist and exercises (Figure 2A). The twist didn’t have any effect on the difference in the sub-tendons strain: the trend of both peak and average strains for each sub-tendon among the rehabilitation exercises was the same when comparing the three types of twist.

## 4 Discussion

This study assessed the effect of different types of rehabilitation exercises on tendon strains, and ranked them based on the average strain in the mid-portion of the tendon (Figure 2B). Although the tendon strains follow changes in the muscle forces, this study shows the potential to use subject-specific FE models to provide information on the specific location of high strains within the free tendon and determine the effect of different twist types. Based on the ranking of the rehabilitation exercises, to gradually increase the tendon strain, it should be recommended to start with heel walk, followed by toe walk, unirise, unidrop and finally to unidrop bent. The type of tendon twist influences the peak strains but not the average. Type I consistently showed highest peak strains among the five rehabilitation exercises.

The original eccentrics-only approach in Achilles tendinopathy rehabilitation (Stanish et al., 1986) was extended by Alfredson et al. (1998) with the inclusion of eccentric exercises with a knee bent, to activate the SOL muscle more and cause a redistribution of the muscle force. Our results showed that bending the knee not only redistributes the muscle forces but also increased both the peak and average strain in the AT by approximately 2%. In addition, the location of the highest strain also moves from more proximal to the mid-portion. This variety in location of the peak strain might be beneficial for the loading of the tendon. However, from these results it is clear that bending the knee strains the tendon more and might need to be included in a later stage of the rehabilitation program. The eccentrics-only approach has also been enriched by other contraction modes. For example, Silbernagel et al.(2001) recommended the combination of eccentric and concentric exercises. The current study was able to demonstrate that during unirise (concentric contraction), lower average strain are observed compared to unidrop (eccentric contraction). If the rehabilitation aims to minimize tendon strain during the initial phase of treatment, our result suggests that rehabilitation should start with concentric exercises and gradually move towards eccentric exercises. More recently, functional exercises to promote speed and energy storage and release, especially towards the end of the rehabilitation program, were introduced (Mascaró et al., 2018). In contrast to our hypothesis, our results show that functional exercises (walking tasks) developed lower average strains compared to the isolated exercises (heel rise and heel drop), suggesting that these functional exercises could be included early in the rehabilitation program.

Our results also clearly show that the type of exercise influences the location of the peak and average tendon strain: these changes, together with the location of the tendinopathy, could be considered to prescribe the correct exercises. For example, comparing the walking tasks with the isolated extended knee exercises, the location of the peak moves from the mid-portion to the proximal side of the tendon. Furthermore, while the functional exercises and the unidrop bent displayed the highest strains in the SOL sub-tendon, the isolated exercises with extended knees did in the GM sub-tendon. This finding may also explain the occurrence of the failure of a single sub-tendon, resulting in a partial tear in the AT (Śmigielski, 2008).

The complexity of the *in vivo* behavior of the AT is well known. Our results corroborates the non-uniform deformations observed *in vivo* (Bojsen-Møller et al., 2004; Arndt et al., 2012; Franz et al., 2015), demonstrating varying tendon strain distribution during the rehabilitation exercises. Using a subject-specific FE model that contains the sub-tendons structure and their relative sliding, we found that going from concentric to eccentric exercise, the difference in strain increases between sub-tendons, and consequently the non-uniform tissue deformations in the AT increase for the eccentric exercises. This difference in strain between sub-tendons increases even more when the heel drop is performed with a knee bent. Ideally, based on these observations, it would be possible to define which exercises stimulates the intra-tendon sliding.

The AT twist has been listed as a contributing factor in Achilles tendinopathy (Bojsen-Møller et al., 2004), but little is known since the twist cannot be quantified *in vivo*. By simulating the three twist types, our study showed that the twist of the sub-tendons influences the peak tendon strains and its location, but not the ranking of the exercises or the average strain. Similar to Shim et al. (2018), the current work found that the least twisted geometry showed the highest peak strain. As peak strain might cause micro-damage to the tendon, this finding suggests that Type I may not be an optimal geometry and may place individuals more at risk of injury. In addition, differently than the other twist types, the highest strain for this tendon type is located in the mid-portion of the tendon for some exercises, which is also the most frequent location of tendon thickening and pain. Unfortunately, Type I is also the twist which most commonly occurs in the individuals (Edama et al., 2015; Pękala et al., 2017). Furthermore, our results showed that the average strain is not influenced by the twist, since the three twisted models showed very similar values of the average strain in the mid-portion, for the different rehabilitation exercises. It is known from previous literature (Pizzolato et al., 2019) that the AT can experience positive adaptation when exposed to strains within a specific range (Wang et al., 2015) or “sweet spot ” The identified “sweet spot” to maintain and promote tendon health (Pizzolato et al., 2019) is 5%–6% tendon strain. Based on our average strain results, the unirise and unidrop exercises fall within this range. However, caution must be taken when interpreting these results, considering the complicated Achilles sub-tendon structure and the above-mentioned sliding mechanism. The inter-individual differences in triceps surae force distribution (Crouzier et al., 2020) and the AT material properties (Yin et al., 2021) need to be taken into account when targeting the “sweet spot.” All these parameters will inherently generate different internal tendon strain distribution. However, to our knowledge, there are no studies investigating strains yet, taking into account the inter-individual differences in Achilles tendinopathy patients. FE modeling represents a promising method for estimating AT strain, as it allows to consider morphological and material properties of different individuals. A future challenge would be to design personalized exercise programs to promote tendon strain within the “sweet spot,” based on FE models results. In this way, the clinical efficacy of exercise-based rehabilitation should improve.

There are some limitations of this study. First, we didn’t include subject-specific material properties and friction between sub-tendons. To answer the current research question, this didn’t seem crucial. However future developments of the FE model will need to take subject-specific material properties and friction into account. Secondly, the ranking of the rehabilitation exercises was solely based on one subject. As such, caution is required in interpreting the outcome since different geometry and exercise executions may influence the AT strains. Therefore, a more comprehensive study including patients with tendinopathy is needed to confirm our findings and providing general guidelines for the prescription of the rehabilitation exercises. However, the study showed the potential of the application of FE models for estimation of AT strains, taking into account a subject-specific geometry. Indeed, since the AT behavior is complex and affected by individual tendon morphology (Yin et al., 2021), a subject-specific morphology should be preferred rather than a general one when modelling the AT for evaluation of the strains.

In conclusion, the AT models developed in this study were able to quantify tendon strain distribution during different type of rehabilitation exercises for Achilles tendinopathy, and the impact of the twisted geometry. Based on average tendon strain in the mid-portion, the rehabilitation exercises ranking was independent of the AT twist. While heel walk developed the lowest average strain, unidrop bent exhibited the highest average strain in the mid-portion of the AT. The eccentric unidrop exercise resulted in higher peak and average strain compared to concentric unirise, for all the three models. The isolated exercises (unirise and unidrop) presented higher average strains compared to the functional exercises (walking tasks). The amount of twist influences the peak strains but not the average. Type I consistently showed highest peak strains among the five rehabilitation exercises. This study was a first step towards future work to design biomechanically informed rehabilitation protocols and provide clinicians with a better guide to prescribe rehabilitation exercises based on their impact on the AT strain.

## Data Availability

The raw data supporting the conclusion of this article will be made available by the authors, without undue reservation.
