# Supplementary material for: Subject-Specific 3D Models to Investigate the Influence of Rehabilitation Exercises and the Twisted Structure on Achilles Tendon Strains
Source: Front Bioeng Biotechnol. 2022 Jul 6;10:914137. doi: 10.3389/fbioe.2022.914137 (PMC9299361; doi:10.3389/fbioe.2022.914137)
Supplement: Supplementary file 1 [file DataSheet1.docx]

Supplementary Material

# Supplementary Data

The values of the material properties that were used for the FE models are the following: C_1_=46.52 MPa, C_3_=19.4 MPa, C_4_=26.80, C_5_=928 MPa and C_2_=0 to describe a Neo-Hookean material. $\lambda_{m}$ was set to 1.03.

# Supplementary Figures and Tables

## Supplementary Figures


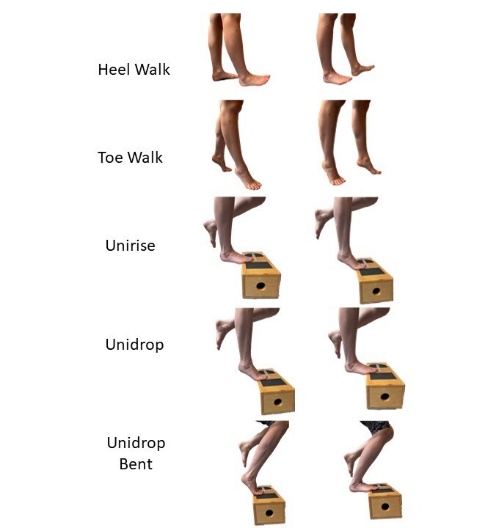


**Supplementary Figure 1.** Execution of the five different rehabilitation exercises: walking on heels (heel walk), walking on toes (toe walk), unilateral heel rise (unirise), heel drop with extended knee (unidrop) and heel drop with knee bent (unidrop bent).
